# “Hasta que Dios diga ‘sí o no’, pero aquí, en mi casa”: autonomía y cuidados de fin de vida en personas mayores rurales de la Región de Los Lagos, Chile

**DOI:** 10.18294/sc.2026.5969

**Published:** 2026-03-13

**Authors:** Ignacia Navarrete Luco, Alejandra Fuentes-García, Verónica Aliaga-Castillo

**Affiliations:** 1 Magíster en Salud Pública. Estudiante, Doctorado en Antropología y Comunicación, Universitat Rovira i Virgili, Tarragona, España. ignav.lu@gmail.com Universitat Rovira i Virgilu Universitat Rovira i Virgili Tarragona Spain ignav.lu@gmail.com; 2 Doctora en Salud Pública. Profesora asociada, Escuela de Salud Pública, Facultad de Medicina, Universidad de Chile, Centro para la Prevención y el Control del Cáncer (CECAN), Santiago, Chile. alefuentes@uchile.cl Centro para la Prevención y el Control del Cáncer (CECAN) Facultad de Medicina Universidad de Chile Centro para la Prevención y el Control del Cáncer (CECAN) Santiago Chile alefuentes@uchile.cl; 3 Magíster en Bioética. Profesora asociada, Departamento de Kinesiología, Facultad de Medicina, Universidad de Chile, Santiago, Chile. valiaga@uchile.cl Universidad de Chile Departamento de Kinesiología Facultad de Medicina Universidad de Chile Santiago Chile valiaga@uchile.cl

**Keywords:** Envejecimiento, Medio Rural, Fin de la Vida, Etnografía, Chile, Aging, Rural Areas, End of Life, Ethnography, Chile

## Abstract

El envejecimiento y aumento de la esperanza de vida generan desafíos crecientes para los cuidados de fin de vida en la vejez, particularmente en contextos rurales marcados por desigualdades territoriales y sanitarias. Desde la geografía gerontológica, y las nociones de autonomía y agencia de las personas mayores, este estudio se propone generar una comprensión del fin de vida como experiencia vital desde los mundos subjetivos de y con las personas implicadas. Para ello, se realizó un estudio cualitativo, de enfoque etnográfico y estrategia de estudio de caso, en la Región de Los Lagos, Chile, entre 2022 y 2023, que incluyó entrevistas semiestructuradas y observación etnográfica a personas mayores rurales en etapas de fin de vida, las personas cuidadoras y los equipos de salud rural. Los resultados muestran que la permanencia en el hogar constituye un deseo central y organiza los cuidados, sostenidos principalmente por redes familiares feminizadas y por la atención primaria rural. El hogar se transforma en un espacio de cuidado y los equipos de salud cumplen un rol clave en acompañamiento clínico y relacional del fin de vida. Se concluye que los cuidados de fin de vida en la ruralidad requieren enfoques territoriales que reconozcan autonomía en la vejez y las desigualdades estructurales de estos procesos.

## Abstract (en)

Aging and increased life expectancy generate growing challenges for end-of-life care in old age, particularly in rural contexts marked by territorial and health inequalities. From the perspective of gerontological geography and the notions of autonomy and agency of older adults, this study aims to generate an understanding of end-of-life as a lived experience from the subjective worlds of and with the people involved. To this end, a qualitative study, with an ethnographic approach and case study strategy, was conducted in the Los Lagos Region of Chile between 2022 and 2023. This included semi-structured interviews and ethnographic observation of rural older adults in the end-of-life stages, their caregivers, and rural health teams. The results show that remaining at home is a central desire and organizes care, sustained primarily by feminized family networks and rural primary care. The home becomes a space of care, and health teams play a key role in providing clinical and relational support at the end-of-life. It is concluded that end-of-life care in rural areas requires territorial approaches that recognize autonomy in old age and the structural inequalities of these processes.

## Introducción

### Eutanasia y cuidados de fin de vida para poblaciones envejecidas

El envejecimiento poblacional acelerado que experimentan los países de América Latina y el Caribe, incluido Chile, se articula con un perfil epidemiológico dominado por enfermedades crónicas y con el sostenido aumento de la esperanza de vida. Una de sus principales implicancias es que la experiencia del morir se concentra crecientemente en personas de edades avanzadas que viven con comorbilidades y distintos niveles de dependencia funcional. A nivel global y local, las principales causas de morbimortalidad en este grupo corresponden a enfermedades cardiovasculares, determinados tipos de cáncer y patologías neurodegenerativas^1^^,^^2^^,^^3^^,^^4^, configurando trayectorias de enfermedad prolongadas y complejas. La prolongación de la vida ha modificado sustantivamente los patrones de morbimortalidad poblacional, desplazando la muerte hacia edades avanzadas y hacia escenarios de mayor requerimiento de cuidados.

Estos cambios, entre otros, se traducen en un aumento de la dependencia de la población mayor que requerirá asistencia y cuidados especializados, en escenarios sanitarios con limitadas capacidades para hacer frente a sus demandas^5^, especialmente en contextos de alta desigualdad social, como ocurre en los países de América Latina y el Caribe. En situaciones de terminalidad, las personas mayores suelen requerir cuidados de largo plazo y cuidados paliativos orientados a aliviar el dolor y el sufrimiento asociados a la enfermedad avanzada^4^^,^^6^^,^^7^. A nivel mundial, se estima que cerca de 40 millones de personas requieren de estos cuidados; sin embargo, solo un 14% accede efectivamente a ellos, evidenciando una brecha estructural entre las necesidades de la población y las respuestas de los sistemas de salud y las políticas públicas^8^.

Los cuidados paliativos son un enfoque integral que busca mejorar la calidad de vida de las personas que enfrentan enfermedades graves o potencialmente mortales. Su objetivo es aliviar el sufrimiento mediante la identificación precoz y el tratamiento del dolor y otros problemas físicos, psicosociales o espirituales^8^. Los cuidados de fin de vida, por su parte, suelen considerarse como el período final dentro de los cuidados paliativos. Buscan brindar el máximo confort y soporte en los últimos días o meses de vida de las personas. De este modo, los cuidados paliativos y de fin de vida se articulan para garantizar la continuidad de los cuidados, especialmente en etapas terminales^9^.

Se reconoce la importancia de combinar cuidados paliativos de alta calidad con un respeto por la autonomía de la persona y su derecho a la intimidad. La relación entre los cuidados de fin de vida y las decisiones de fin de vida, entre estas, la eutanasia, se establece a través del respeto a la autonomía y la capacidad de agencia de las personas, la consideración del marco legal vigente y la búsqueda de una muerte digna como un derecho^10^. 

La autonomía y la agencia de las personas mayores en relación con los procesos de fin de vida son centrales en el campo de la gerontología y la bioética, en cuanto remiten a la capacidad de tomar decisiones informadas sobre los cuidados, el lugar y las condiciones en que desean transitar esta etapa vital^11^^,^^12^. En los países y estados donde la eutanasia y el suicidio asistido están regulados, quienes más solicitan estas prácticas son personas mayores^13^. No obstante, en América Latina, el ejercicio efectivo de dicha autonomía se ve frecuentemente tensionado por estructuras familiares, normas culturales y limitaciones de los sistemas de salud, que tienden a desplazar la voluntad individual hacia decisiones asumidas por otros actores^14^. A ello se suma una situación de vulnerabilidad aumentada, asociada a prejuicios etarios y a un imaginario social que presupone la pérdida progresiva de la capacidad de autodeterminación en la vejez^15^, incluyendo las decisiones relativas a la atención en salud y al fin de la vida. La Convención Interamericana sobre la Protección de los Derechos Humanos de las Personas Mayor^16^ constituye un referente normativo clave al establecer obligaciones para garantizar la autonomía y dignidad en la vejez, cuestionando explícitamente las concepciones culturales que asocian el envejecimiento con la pérdida de agencia.

Pese al acelerado envejecimiento poblacional de Chile, estas problemáticas han sido abordadas insuficientemente tanto en el ámbito académico como en el de las políticas públicas^17^, lo que ha contribuido a postergar debates complejos en torno a las necesidades diferenciales que emergen en los procesos de morir en la vejez, así como a la forma en que estos son gestionados institucionalmente. Si bien la eutanasia no es actualmente legal en el país, se han producido avances en la discusión legislativa y existe un marco normativo que reconoce el derecho a rechazar o limitar tratamientos médicos en determinadas condiciones. Dichas transformaciones normativas coexisten con un desarrollo incipiente de políticas sanitarias orientadas a los cuidados de fin de vida de las personas mayores, las que continúan siendo abordadas tangencialmente por los programas existentes. Esta brecha resulta crítica en contextos rurales, donde las barreras de acceso a la atención sanitaria y a los cuidados formales profundizan la dependencia de arreglos de cuidado no remunerados, de carácter familiar y marcadamente feminizados^18^^,^^19^^,^^20^.

### Envejecimiento, territorios y experiencias de fin de vida

El envejecimiento poblacional presenta una expresión territorial diferenciada central para comprender las condiciones en que se desarrollan los procesos de fin de vida. Si bien la mayor parte de la población mayor reside en áreas urbanas, los territorios rurales concentran proporcionalmente una alta presencia de personas mayores y exhiben dinámicas de envejecimiento más aceleradas^21^^,^^22^. En Chile, el índice de envejecimiento en zonas rurales supera al de las áreas urbanas, con 139,4 personas mayores de 60 años por cada 100 menores de 15 años, frente a 100,2 en contextos urbanos según datos de 2024^23^. En localidades rurales se observa una proporción de hogares principalmente conformados por personas mayores y de jefaturas de hogar envejecidas, dando lugar a núcleos familiares altamente envejecidos^24^. Estos procesos se explican por las migraciones internas de generaciones jóvenes hacia las ciudades, la expansión de actividades extractivas y agropecuarias, y fenómenos de gentrificación rural o migración por amenidad, que han contribuido al despoblamiento sostenido y al envejecimiento de estos territorios^25^^,^^26^.

Desde la geografía gerontológica^22^ se ha destacado que los territorios rurales no deben ser comprendidos únicamente como escenarios de carencia, sino como espacios socioculturales heterogéneos, atravesados por transformaciones estructurales que inciden en las trayectorias vitales y en las experiencias cotidianas de las personas mayores^22^. En este marco, las personas mayores son reconocidas como agentes activos en los procesos de cambio de sus comunidades, portadoras de posiciones relacionales y simbólicas relevantes en los núcleos domésticos y comunitarios, incluso en contextos de pérdida de movilidad o aumento de dependencia funcional^27^. En relación con los cuidados de fin de vida, estos procesos movilizan a familias, actores comunitarios e instituciones locales, reconfigurando las relaciones sociales y las formas de provisión de cuidados y prestaciones sanitarias. Así, el fin de la vida no solo interpela la autonomía individual, sino que repercute en las dinámicas y prácticas de las comunidades rurales en su conjunto.

Las poblaciones rurales envejecidas enfrentan profundas desigualdades en salud, asociadas en parte a la centralización urbana de los servicios sanitarios^20^, tanto públicos como privados^4^. En comparación con las áreas urbanas, la disponibilidad de centros de atención primaria, el acceso oportuno a servicios hospitalarios, farmacias y sistemas de transporte público es significativamente menor, fenómeno que se reproduce en distintos países de América Latina^18^. Estas desigualdades se inscriben en procesos históricos de exclusión socioterritorial, particularmente acentuados en países con altos niveles de centralización político-administrativa, como Chile^25^. 

Las brechas de acceso a la atención sanitaria y a los cuidados formales generan respuestas locales de carácter familiar-comunitario para cubrir los procesos de salud/enfermedad/atención de las personas mayores rurales^18^^,^^19^^,^^27^^,^^28^. En este contexto, la provisión de cuidados depende en gran medida de la capacidad económica y organizativa de las familias, profundizando desigualdades socioeconómicas y territoriales. Por ejemplo, en México se ha reportado que este gasto económico es más elevado en personas mayores de 90 años que residen en zonas rurales, en comparación con otros grupos etarios mayores de 60 años^29^. Esta situación refleja, por una parte, una precarización institucional de la atención sociosanitaria de las poblaciones rurales envejecidas y, por otra, la articulación y despliegue de estrategias comunitarias para la atención y cuidados de personas mayor^19^. 

Aunque la producción de evidencia sobre salud y cuidados en poblaciones rurales envejecidas ha sido limitada en mostrar la complejidad de las experiencias de salud, enfermedad y cuidado de las personas mayores rurales, así como de las respuestas familiares, comunitarias e institucionales que se articulan en torno al fin de la vida^18^^,^^21^^,^^30^, el corpus investigativo ha mostrado un crecimiento sostenido en los últimos años^28^^,^^31^^,^^32^^,^^33^. Esta literatura coincide en identificar brechas estructurales persistentes en la implementación de servicios de salud y en el acceso a cuidados paliativos y de fin de vida en territorios rurales^19^^,^^22^^,^^34^, subrayando la necesidad de profundizar enfoques territoriales que permitan comprender las condiciones concretas en que las personas mayores transitan el final de la vida.

### Políticas de cuidado del final de la vida en Chile: avances normativos y brechas territoriales

En Chile, las políticas e intervenciones orientadas al cuidado del final de la vida son incipientes y se gestionan principalmente desde el nivel de atención primaria. Entre las iniciativas vigentes, el Programa de “Atención Domiciliaria para Personas con Dependencia Severa” (PADPDS) del Ministerio de Salud^35^ brinda atención a personas con dependencia severa de todas las edades, incorporando componentes promocionales, preventivos y curativos, y acompañando tanto a las personas en situación de dependencia como a las personas cuidadoras. Este programa carece de directrices específicas orientadas al cuidado de fin de vida, pese a que en la práctica los equipos atienden con frecuencia a personas en etapas terminales.

Un avance relevante fue la promulgación de la Ley 21375 en 2021, que consagra los cuidados paliativos y los derechos de las personas con enfermedades terminales o graves^36^, así como su implementación a partir de 2023 mediante el Programa de Cuidados Paliativos Universales^2^. Esta política amplió el acceso a prestaciones farmacológicas y no farmacológicas, promoviendo un enfoque integral del cuidado y reconociendo explícitamente a las personas mayores como un grupo con alta probabilidad de requerir estos servicios, en función de los perfiles epidemiológicos actuales. Su implementación supuso un cambio sustantivo respecto de la situación previa, en la que los cuidados paliativos estaban restringidos a personas con patologías específicas en etapas avanzadas, como el cáncer^37^, las demencias^38^, la enfermedad renal crónica en etapas 4 y 5 y la enfermedad de Parkinson^39^^,^^40^.

Actualmente se han impulsado dos proyectos de leyes en Chile en los que los cuidados de fin de vida y, eventualmente la eutanasia, podrían ampararse como parte de los derechos de las personas mayores. Por una parte, la Ley Integral de Personas Mayores y Promoción del Envejecimiento Digno, Activo y Saludable^41^, pendiente de ser promulgada, en su artículo 2 señala la “dignidad, independencia, protagonismo y autonomía de la persona mayor” y extiende estas indicaciones para las decisiones de fin de vida. Por otra parte, el proyecto de ley que reconoce el derecho al cuidado y crea el Sistema Nacional de Apoyos y Cuidados^42^, sobre la base del reconocimiento del cuidado como derecho social y humano, y con objetivos de mejoramiento de la calidad de vida tanto de quienes reciben cuidados, como de quienes lo proveen. Los avances están enfocados en aumentar las coberturas actuales y la generación de nuevos programas y beneficios sociales.

Estos avances normativos coexisten con una limitada producción de evidencia empírica que permita comprender cómo se desarrollan concretamente las atenciones y cuidados de fin de vida que recibe la población mayor, así como la capacidad del sistema de salud para responder a sus necesidades en contextos diversos^7^^,^^30^. Esta brecha resulta particularmente marcada en el caso de las personas mayores que habitan territorios rurales, donde las condiciones de acceso, la disponibilidad de servicios y la organización de los cuidados difieren sustantivamente de los contextos urbanos, profundizando desigualdades sociosanitarias preexistentes.

En este escenario, el presente artículo -basado en la tesis de Magíster en Salud Pública (Universidad de Chile) de la primera autora, titulada “Cuidados de fin de vida a personas mayores en localidades rurales de la provincia de Llanquihue, Región de Los Lagos, Chile” - busca contribuir, desde una perspectiva territorial situada, a la comprensión de los cuidados de fin de vida de personas mayores que habitan localidades rurales de la provincia de Llanquihue, Región de Los Lagos. A partir de la geografía gerontológica^22^^)^ y la autonomía y la agencia de las personas mayores^27^, el estudio se propone visibilizar un punto ciego de la política sociosanitaria chilena: la ausencia de enfoques que integren explícitamente las desigualdades territoriales y las condiciones concretas en que los cuidados de fin de vida se despliegan, aportando evidencia empírica relevante para el debate sobre envejecimiento, cuidados y justicia sociosanitaria en los proceso de fin de vida.

## Materiales y métodos

Se realizó una investigación cualitativa de tipo descriptivo-exploratorio, bajo un enfoque y método etnográfico, y con una estrategia de estudio de caso, para generar una comprensión del fin de vida como experiencia vital desde los mundos subjetivos de y con las personas implicadas en el fenómeno a estudiar, es decir, desde una perspectiva emic^43^. La característica descriptiva-exploratoria^44^ permitió indagar en un fenómeno poco estudiado como son los cuidados de fin de vida de personas mayores rurales, describiendo cómo se despliegan y organizan los cuidados en este proceso vital en territorios y casos particulares. La estrategia muestral se definió según los parámetros del estudio de caso^45^, para comprender en profundidad las dinámicas internas y contextuales de temas contemporáneos poco tratados. El estudio de caso busca abordar de forma intensiva una unidad^46^, siendo esta una persona, una familia, un grupo, una organización o una institución^47^. Para la producción de información combina distintas técnicas, tanto de documentación, realización de entrevistas, observación etnográfica y/o aplicación de métodos biográficos. Con relación a ello, se trabajó con una muestra de tipo teórica^44^ con tres casos compuestos por la persona mayor y la persona cuidadora, y los equipos de salud del PADPDS. La convocatoria y reclutamiento de las personas participantes se realizó a través de los equipos del PADPDS en comunas rurales de la provincia de Llanquihue, Región de Los Lagos, con quienes se seleccionaron los casos conjuntamente. Los criterios de inclusión fueron: personas mayores inscritas en el PADPDS de este territorio y cuya expectativa de vida fuese de seis meses o menos, junto a las personas cuidadoras y los equipos de salud que realizan prestaciones en el programa, con una experiencia laboral en el propio programa de al menos 12 meses. El trabajo de campo se desarrolló entre diciembre de 2022 y octubre de 2023. 

Se realizaron entrevistas individuales semiestructuradas a personas cuidadoras y a personal profesional y/o técnico de salud implicado en el cuidado de fin de vida de una persona mayor. Las entrevistas se ejecutaron en sesiones diferenciadas. Se realizaron entrevistas por separado a personas cuidadoras y equipos de salud, para lo cual se elaboró un guion temático para personas cuidadoras y otro para equipos de salud, obteniéndose información relacionada con las dimensiones sobre la organización social del cuidado, significados sobre el fin de vida de personas mayores rurales y reflexiones sobre este proceso en contextos rurales del sur austral. Dicho guion fue trabajado en pautas de entrevistas semiestructuradas, incorporando además la posibilidad para abordar temas no previstos por la investigadora. También se diseñó una pauta de observación etnográfica que permitió guiar el registro de notas de campo, facilitando la sistematización del material empírico. Las entrevistas fueron grabadas en audio digital y fueron transcritas. La observación etnográfica se realizó en el marco de las visitas y las sesiones de entrevistas en el hogar, donde se entablaron relaciones con las personas participantes y se desarrolló una observación intensiva, rigurosa y adaptada a los contextos domésticos, significados de fin de vida y de cuidados, así como relaciones sociales entre equipos de salud, personas cuidadoras y persona mayor. El proceso implicó un “estar allí” y compenetrarse con la realidad a estudiar. 

Con relación al fin de vida de personas mayores como tema de estudio, se acoge lo que Brage^48^ nombra como una etnografía de alta complejidad. En su investigación doctoral, donde trabajó los itinerarios terapéuticos con madres e infancias con cáncer, la autora reflexiona cómo la actividad etnográfica le resultó “una experiencia compleja, solitaria y desgastante”^48^, donde la inmersión en el campo le llevó a atravesar estados de ánimo diversos, así como desánimos. Comprender las emociones producidas durante el trabajo de campo -como parte de la reflexividad del método etnográfico-, es clave para entender procesos de investigación en los que, al compenetrarse con el sufrimiento ajeno, resulta inevitable no afectarse en ello. Sumergirse en la muerte, preguntar por ella -tanto en cómo es concebida, cómo es imaginada y cómo es deseada-, reviste una mezcla de incomodidad y de coraje por parte de quien pregunta/observa un tema escondido y temido socialmente. Asumiendo que la reflexividad es una cualidad encarnada en el trabajo etnográfico, esto no significa que el contar con esta herramienta simplifique la experiencia, más bien, la puede tornar compleja. Pero dicha afectación no solo corresponde a una emocionalidad individual y, por ende, puede englobarse en el fenómeno colectivo que es cuidar y acompañar los procesos de fin de vida. 

Se utilizó un análisis etnográfico para la comprensión y tratamiento del fenómeno estudiado, reuniendo el material empírico en un corpus que permitiera la descripción densa^49^ por cada estudio de caso. La información producida en entrevistas semiestructuradas y registro de observación etnográfica fue introducida y sistematizada en el software Atlas Ti. v23. El análisis fue temático^50^ y la codificación fue deductiva-inductiva según los objetivos e instrumentos del estudio, permitiendo organizar la evidencia^44^, identificar las citas significativas y aquellos elementos emergentes de análisis. Se generaron 22 códigos, reunidos en 4 grupos de códigos (fin de vida; territorio; salud rural; y organización social del cuidado). Luego, se construyó un relato etnográfico por cada estudio de caso a modo de presentación de resultados, donde se describió el contexto socioterritorial de la comuna de estudio, las características comunitarias y familiares del caso estudiado, y las reflexiones de los equipos de salud rural sobre las desigualdades en salud y la organización social del cuidado para aquellas personas en procesos de fin de vida. En este relato se generó una articulación etnográfica^51^, en la cual la producción empírica de los datos dialoga con el contexto territorial, sociocultural y sanitario de cada caso de estudio, además de las perspectivas teóricas del estudio, como son la gerontología geográfica y la autonomía y agencia de las personas mayores. Se privilegió la creación de textos polifónicos, en los que están presentes las voces de las personas cuidadoras, las personas mayores, los equipos de salud y la investigadora. 

Esta investigación se desarrolló en la Región de Los Lagos, caracterizada por su amplitud geográfica (48 mil km^2^), una alta dispersión poblacional y desmembramiento territorial (con presencia de ríos, lagos, fiordos, sistemas insulares y archipelágicos). Las condiciones climáticas adversas, marcadas por alta pluviosidad anual y centralización de servicios básicos en la capital regional de Puerto Montt, singularizan la experiencia del envejecer rural^32^.

En este artículo se presenta uno de los tres casos investigados, correspondiente a un hombre nonagenario y su núcleo de cuidados familiares y sanitarios, ya que ilustra la capacidad de autonomía y agencia en los procesos y cuidados de fin de vida en contextos rurales. El relato está escrito en primera persona por la primera autora del artículo.

### Aspectos éticos

Esta investigación fue aprobada en diciembre del 2022, mediante el Acta No. 179 por el Comité de Ética de Investigación en Seres Humanos de la Facultad de Medicina, Universidad de Chile, acreditado por la resolución exenta No. 2413441782 del 14 de noviembre de 2024. Basándose en los documentos “Lineamientos para la evaluación ética de la investigación en Ciencias Sociales y Humanidades”^52^ y en los requisitos éticos^53^, el estudio dio cumplimiento a los estándares éticos de la investigación social con seres humanos. Se realizó un proceso de consentimiento informado con todas las personas participantes, indicando aspectos de voluntariedad, riesgos, beneficios, devolución de resultados, así como contacto de la investigadora principal. Se adecuaron los documentos a las particularidades de cada informante (persona mayor, persona cuidadoras y equipos de salud). El material empírico producido fue almacenado en la nube de datos de la investigadora principal. Se han utilizado pseudónimos para la identificación de las personas participantes, a modo de resguardar la confidencialidad y el anonimato.

## Resultados

Anselmo, de 94 años, en el momento del trabajo de campo residía en el sector “El Bosque”, de la comuna de Cochamó, ubicada en el sector oriental de la Región de Los Lagos, colindante con la frontera argentina. Es un territorio de 3.910 km^2^, con una población de 4.199 personas^54^, con una densidad poblacional de 1,07 hab/km^2^. Tiene características cordilleranas y de ruralidad de alto aislamiento, dificultando la implementación y acceso de programas públicos. La red de atención primaria está compuesta por un centro de salud familiar y nueve postas rurales. No existen prestadores privados en este sector. 

Para este caso específico, el trabajo de campo fue realizado entre diciembre de 2022 (notas etnográficas con Anselmo y su familia, y entrevista semiestructurada con médico de la posta rural de Cochamó) y enero de 2023 (notas etnográficas y desarrollo de entrevista semiestructurada con Anselmo y su hija cuidadora). No se pudo concretar la última visita etnográfica donde se observaría la prestación del PADPDS en el domicilio de Anselmo, ya que falleció en febrero de 2023. 

### “Estar cuando Dios diga ‘sí o no’, pero aquí, en mi casa”

Él desea conversar. A sus 94 años y con un cáncer pulmonar avanzado, persiste en hablar, a pesar de una falta de aire que le cansa e imposibilita llevar diálogos fluidos. Por momentos tenemos que pausar la entrevista y esperar hasta que recupere el aliento. Para ayudarlo, Adela -una de las hijas cuidadoras- agarra un pedazo de cartón -el *“soplador”*, como han titulado a esa inventiva de cuidado-, moviéndolo enérgicamente de arriba hacia abajo para *“hacer aire”*, permitiéndole a su nonagenario padre refrescarse. Es un día brillante de sol estival y, en el valle de Cochamó, ese calor se extrema e intensifica al interior de casas añosas construidas de madera nativa. Dejamos la puerta abierta, entrando y saliendo a su antojo un perro mestizo pequeño, que se golpea contra las paredes de la casa. Está ciego y viejo, *“como yo”*, señala Anselmo riendo.

La familia vive en una ladera de cerro del sector El Bosque, distante a unos 5 kilómetros del área comercial y administrativa de Cochamó. Actualmente se ha convertido en un atractivo turístico, ya que en lo alto de uno de los montes instalaron un columpio para balancearse y apreciar el estuario del Reloncaví. Esta afluencia mayor de público no es sinónimo de mejoras en la infraestructura vial rural del lugar. Como se señala desde la gerontología geográfica, el desarrollo rural contemporáneo beneficia y robustece un desarrollo exógeno y privado que no necesariamente genera un desarrollo local^22^. Para llegar al hogar de Anselmo, me lleva Ana, autodenominada como la cuidadora principal, quien vive a unos 300 metros de la casa de su padre. Llegar a la casa de Ana no es simple, solo es posible a través de caminata, y la experiencia de ese trayecto permite una inmersión en las barreras de acceso de los cuidados sanitarios en territorios rurales. El camino es angosto y barroso y, por algunos momentos, las arboledas oscurecen el trayecto. Con la guía de Ana, ingresamos por un portón de madera y vamos subiendo por huellas llenas de vegetación sureña.

Del matrimonio nuclear nacieron ocho hijos e hijas -uno de ellos, Amado, falleció de cáncer en 2022-, y Ana, Adela y Andrea son claves en el auxilio y sostén de la vejez de su progenitor. Anselmo ha vivido solo desde que enviudó hace unos años atrás. Esta reconfiguración habitacional unipersonal es sopesada con estrategias familiares del mundo rural como, por ejemplo, que las generaciones más jóvenes construyan casas aledañas al hogar del progenitor. Ana y Andrea lo rodean en ese terreno en pendiente, cada una en sus respectivas casas. Además de la presencia de estas hijas, por temporadas se queda en la casa Adela, de 57 años, que vive en Punta Arenas, la ciudad más austral de Chile. 

Hombre gaucho y arriero, Anselmo despliega un uso fonético en las formas de hablar solo rastreable en la Patagonia del sur austral, y que es parte del habitar fronterizo trasandino. La presencia de Anselmo está envuelta en elegancia cordillerana. Viste de traje, pañuelo estampado anudado en el cuello, y la infaltable boina gaucha que enaltece las cabezas de los hombres del sector. Ese decoro esconde una corporalidad delgada y huesuda, la que atiende para evitar heridas y dolores con un cojín antiescaras, al que llaman *“picarón”* [masa dulce preparada en Chile], por su forma en “O”. Vive con una ceguera casi total y, en la interacción del diálogo, se olvida porque pareciera que te mira, observa. En esa mirada ciega hay autonomía y dignidad. La primera visita etnográfica fue a fines de diciembre de 2022. Para esa fecha se estaba recuperando de una fuerte neumonía, tratada con antibióticos, oxigenoterapia e intensos cuidados del equipo de salud y de sus hijas. La visita etnográfica y la conversación giraron en torno a la organización de los cuidados al interior de la familia y el cotidiano rural: cómo es la percepción de la atención del equipo de salud del PADPDS, cuántos animales hay y algunas anécdotas del zorro que baja del monte para matar aves. No hay un orden lineal conversacional, sino que los contenidos se intercalan entre sí, creando una imagen de aquellos cuidados de la vida que le queda y de la muerte que le espera.

El fallecimiento de su hijo fue un golpe emocional arduo de sobrellevar. En la interacción etnográfica, Adela cuenta que éste fue un evento que marcó la aceleración del proceso de dependencia y enfermedad de Anselmo, porque antes, estaba bien. Para graficar este estado previo, las mujeres cuentan orgullosas que Anselmo fue junto a Amado al pueblo -es decir, a Cochamó- en caballo. Ante mi sorpresa, que pudo percibir Anselmo, ¿cómo una persona ciega puede andar en caballo?, el nonagenario aclaró *“el caballo lo guía a uno”* (notas etnográficas, diciembre del 2022). Pero falleció su hijo y decayó. Observando ya la dificultad para respirar, desde el PADPDS llevaron a *“arturito”*, apodo otorgado al tanque de oxígeno por su semejanza con el robot de la película Star Wars. Dado el crítico estado en que se encontraba, las hijas cuentan que, tras la visita médica al hogar, se pensó en ingresar a Anselmo en el hospital de alta complejidad de Puerto Montt [capital regional de Los Lagos]. Pero él no quiso: *“Yo no, porque iba a quedar abandonado, y ya no es lo mismo, ir a otra casa”*, opina Anselmo respecto a lo que significa e implica hospitalizarse, el abandono y estar en un lugar diferente a su mundo-hogar. Este parecer puede que se construya en procesos reflexivos sobre experiencias familiares previas, vinculadas a recintos hospitalarios. De hecho, su esposa estuvo internada en el hospital de Puerto Montt antes de morir; como él dice: *“fue y no volvió más”*. 

Al observar el delicado estado de salud de Anselmo, el médico de la posta rural comenzó a preparar a las personas de la familia sobre el proceso de fin de vida del nonagenario, anticipándoles las situaciones que podrían ocurrir y ayudándolas en la toma de decisiones:

“*Es ir preparando a la familia siempre para lo que va a ir ocurriendo, que ellos sean los dueños de la información y que ellos decidan qué hacer con ello. Siempre revisando lo que sabe la persona de lo que le está pasando, lo que sabe la familia de lo que le está pasando a su familiar*”. (Médico posta rural, Cochamó, entrevista diciembre de 2022)

Ante la posibilidad de un aumento del dolor, se puede activar la entrega de medicamentos para su manejo desde la atención primaria -el más usado, el parche de morfina-, ya que por su perfil de paciente oncológico estaba disponible el acceso a este tipo de fármacos. A la fecha del trabajo de campo en Cochamó (diciembre de 2022 y enero de 2023), aún no se había implementado el Programa de Cuidados Paliativos Universales.

La atención a la neumonía fue en domicilio, por el equipo del PADPDS de la posta rural. Los exámenes que mostraron el diagnóstico de cáncer pulmonar fueron tomados en un centro de salud privado en Puerto Montt. Ante la posibilidad de seguir tratamientos de alta complejidad u hospitalizarse, Anselmo tenía su decisión clara: no seguiría una terapia invasiva. La familia entendió la voluntad y autonomía en la toma de decisiones de su padre:

“*…es que igual hay que respetarlo a él, porque llevarlo a la fuerza al hospital no, porque ya lo lleva bien, porque él está consciente, no se le puede obligar. Sin su consentimiento uno no puede hacer*”. (Adela, hija de Anselmo, entrevista enero de 2023)

La palabra “cáncer” se enuncia poco, por las muertes recientes familiares cuyo diagnóstico fue esta patología. La aceleración de la enfermedad de Anselmo ha hecho que las mujeres de la familia se transformen en expertas en cuidados básicos de enfermería. Pastillero, inhalador, cama clínica, oximetría de pulso y administración de oxígeno, entre otros, son ya conocidos y manejados técnicamente. Le pido a Adela que me muestre los insumos y trae el saturómetro. Para ilustrar el uso, hacemos la prueba con Anselmo. Su saturación es de 93% y la frecuencia cardíaca de 92%. *“Buenos indicadores”,* dice, *“cuando la saturación está bajo del 90% hay que preocuparse”.* Recientemente, desde la municipalidad les llevaron el colchón antiescaras, al que se le debe regular el aire. Aún están aprendiendo cómo usarlo. Alzando la voz, Adela le pregunta a Anselmo: *“¿Cómo durmió papá?”,* y con burla sureña, el nonagenario contesta: *“Catay* [sic, expresión local de burla o asombro]*, encima de un piedrero”.*

El colchón debe inflarse de acuerdo con el peso de quien lo usa, de lo contrario se endurece demasiado y genera un efecto sensorial de rebote. Por las noches, las hijas se turnan para acompañar a Anselmo mientras duerme, ya que a veces se ahoga y necesita oxígeno. Observar los insumos médicos, así como los conocimientos técnicos asociados a su uso, revelan cómo el hogar se transforma en un espacio de cuidado especializado, donde las tecnologías sanitarias se insertan en la cotidianeidad, posibilitando la permanencia en casa. Este aspecto permite materializar el deseo y la voluntad de permanecer en el hogar, aun en un contexto de marcado aislamiento rural. Se visibiliza la posibilidad de sostener cuidados complejos o de mediana complejidad en el hogar, lo que genera oportunidades de diseñar políticas de cuidados, especialmente de fin de vida, que incorporen a las familias y comunidades^4^^,^^18^^,^^21^.

En el hogar del nonagenario conviven distintas creencias, prácticas de cuidado y curación. Se profesa la fe católica y se usan hierbas locales. En las descripciones del sistema médico local, se cuela la presencia del equipo del PADPDS. El vínculo es estrecho, y padre e hija reconocen el trabajo de las personas del equipo profesional y técnico que los visitan. Con un tono confesional, Anselmo señala: *“sabe qué, le voy a decir algo. No sé por qué, cuando vienen los médicos, me siento mejor”*. La declaración deja entrever una idea: no es el rechazo a la biomedicina, ya que de hecho hay prácticas de este tipo insertas en el cuidado cotidiano en el hogar, sino el rechazo a tener que abandonar el hogar para acceder a tratamientos y cuidados especializados. Porque al hospital, en palabras de Anselmo, *“va a sufrir no más uno”*. Existe una valoración del sistema alópata cuando este se despliega en la ruralidad, activándose una suerte de eficacia simbólica^55^ cuando la atención se realiza en el hogar. En este sentido, Adela agrega:

“*…es mejor que sacarlo, así como está él, es más cómodo absolutamente, más cómodo que vengan a la casa. Cuando los llaman, las chicas [técnicas paramédicas de la posta rural] ahí vienen, se hacen un tiempito, programan ellos su visita*”. (Adela, hija de Anselmo, entrevista enero de 2023)

Hace ocho días acudió el enfermero, para revisar una úlcera por presión que tenía Anselmo, la cual fue tratada con una crema que trajo uno de los hijos y con el uso del nuevo colchón antiescaras. 

La experiencia de enfermedad de Anselmo fue una primera aproximación metodológica para abordar el proceso de fin de vida, que revistió la reflexión de cómo conversar de la muerte con una persona mayor y su familia. Algo de ese conflicto se puede leer en la transcripción de la siguiente entrevista, como también algo de la claridad y simpleza del tema:

-“*Don Anselmo, le quiero hacer una pregunta que puede ser un poco íntima: si usted ha pensado en sus últimos días de vida, ¿le gustaría estar en la casa?*

-*Claro… claro, sí… sí…*

-*¿Usted ha pensado cuál sería su deseo?*

-*Estar aquí en mi casa, y estar cuando Dios diga ‘sí o no’, quiero estar con mis hijos tranquilo aquí, sí*”. (Anselmo, entrevista enero de 2023)

Luego de una hora de conversación, Anselmo ya quiere terminar la entrevista. Al parecer, represento un vínculo con el equipo de salud. Al despedirnos me deja un encargo:

“*Cuando vea a sus colegas, los médicos, dígale que me vengan a ver, no me dejen, los que anduvieron pa’ acá… que me vengan a ver, que no me olviden*”. (Anselmo, entrevista enero de 2023)

Parte de la experiencia etnográfica es el compartir e intercambiar vivencias fuera del marco de la entrevista, técnica conocida como observación participante. En razón a ello, le pido a Adela que me muestre sus animales y parte del terreno familiar. Salimos a recorrer el campo, a mirar las ovejas y apreciar el Estuario del Reloncaví desde el monte de El Bosque. El tiempo fuera de la casa es breve, ya que Anselmo no puede quedarse solo por mucho rato. Cuando Adela viaja desde su hogar en Punta Arenas para pasar una larga temporada en Cochamó, su trabajo son los cuidados de su padre, y se dedica exclusivamente a ellos. Por eso, está alerta a que su progenitor pueda necesitar apoyo o a que le puede ocurrir algo -una caída, por ejemplo- si permanece solo en casa. Se percibe su emoción y tristeza, porque sabe cuál es el devenir de la enfermedad de su papá. Pero también se percibe su felicidad, por poder estar ahí, cuidar y acompañar.

## Discusión

Los hallazgos de este estudio de caso muestran que, en contextos rurales de alto aislamiento, los procesos de fin de vida de personas mayores se configuran a partir de una compleja articulación entre autonomía y agencia individual, arreglos familiares de cuidado, presencia selectiva de la atención primaria de salud y uso cotidiano de tecnologías sanitarias en el hogar. La experiencia analizada evidencia que la permanencia en el domicilio constituye un deseo de Anselmo, y un principio organizador de las decisiones de cuidado, incluso frente a diagnósticos de alta complejidad, como es el cáncer avanzado. Se observa una progresiva transformación del hogar en un espacio de cuidado especializado, sostenido por mujeres de la familia, quienes adquieren competencias técnicas y asumen una alta carga de cuidado ante las limitaciones del acceso territorial a servicios formales. La atención primaria rural es clave en el acompañamiento del proceso de fin de vida, no solo desde una dimensión clínica, sino también relacional y simbólica, al posibilitar decisiones informadas y respetuosas de la voluntad de la persona mayor.

Una de las preocupaciones centrales de este artículo es cómo se atiende el fin de vida de personas mayores que habitan en territorios rurales. Desde la evidencia de los datos que sostienen el mayor índice de envejecimiento en la ruralidad, la aproximación etnográfica permite dar cuenta de esta realidad desde la experiencia vivida y, de esta manera, complejizar el conocimiento situado sobre este fenómeno. 

Los territorios rurales han estado marcados por una baja y fragmentada presencia de políticas e intervenciones estatales, por lo que ante necesidades de salud, se despliegan los cuidados informales^18^, con base en la permanencia de arreglos y acuerdos familiares y comunitarios en los procesos de salud/enfermedad/atención^19^. En el caso de Anselmo, se observa la presencia de una red familiar cuidadora feminizada que cuenta con el apoyo de cuidados formales del nivel primario de atención rural. Por una parte, la información empírica coincide con la evidencia sobre el rol de las mujeres en el cuidado a personas mayores con dependencia^15^^,^^18^, mostrando una extensión de estos cuidados hacia los procesos de fin de vida^56^. Por otra parte, se evidencian las capacidades de los dispositivos rurales para activar atenciones de fin de vida, tales como el suministro de medicamentos, la gestión para la entrega de insumos como el colchón antiescaras o el tanque de oxígeno y la preparación de familiares frente a la sintomatología asociada al fin de vida. Estos recursos permiten la permanencia en el hogar mediante la suerte de una hospitalización domiciliaria, considerada una condición propicia para la preparación y el afrontamiento de la muerte^7^. 

Considerando las distancias simbólicas y de acceso de las poblaciones rurales hacia el sistema hospitalario, se podría interpretar que, en el caso de Anselmo, no se evita este modelo de atención, sino que no desea abandonar su hogar ni su tierra para ser hospitalizado en un centro de salud de alta complejidad. Se puede pensar en la muerte asistida “comunitaria”, en territorios rurales, en tanto existan condiciones que lo permitan como son, entre otros, presencia de una atención primaria de salud fortalecida, cohesión comunitaria y redes de cuidados y apoyos. Otro de los aspectos significativos, es el respeto a la autonomía de Anselmo en las decisiones sobre su enfermedad, tanto por parte de sus hijas cuidadoras como del equipo de salud rural. El nonagenario decide no realizar un tratamiento oncológico y cursar la enfermedad en su hogar, en su tierra. Permanecer en este espacio significa, también, permanecer en un territorio que define su identidad y cultura.

Para el fortalecimiento de los estudios sobre fin de vida en clave interdisciplinar y en contextos situados latinoamericanos -caracterizados por una alta ruralidad y cuidados marcados por el familiarismo-, es relevante visibilizar los desafíos en el estudio de estos procesos vitales. El conocimiento profundo del proceso de fin de vida es de difícil acceso, en tanto se desarrollan en espacios íntimos familiares, a los que en general solo se admite el ingreso de profesionales o técnicos de salud u otros agentes comunitarios significativos. Ello puede implicar barreras en la investigación al momento de indagar y observar los procesos de terminalidad que anteceden a la muerte. 

Cabe señalar que el caso expuesto no es representativo de los cuidados de fin de vida en la ruralidad ya que, en la intersección de ruralidad y alta demanda de cuidados, predominan las inequidades y desigualdades en salud. El caso de Anselmo refleja una experiencia excepcional en la que se observa cohesión entre familias y equipos de salud rural, acceso a insumos e implementos médicos, y respeto por las decisiones y voluntades en torno a la propia vida durante la vejez avanzada.

## Conclusiones

El caso de Anselmo permite visibilizar cómo, en contextos de envejecimiento poblacional acelerado y de ruralidad marcada, es posible articular cuidados de fin de vida que integren recursos biomédicos, redes comunitarias y saberes familiares, todo ello sostenido en el respeto por la autonomía y la dignidad de la persona mayor^16^. Lejos de constituir la norma, esta experiencia representa una excepción dentro de un escenario en el que predominan inequidades en salud y brechas de acceso a cuidados paliativos en la ruralidad latinoamericana. Sin embargo, muestra con claridad que la conjunción de dispositivos de salud rurales, el intercambio de conocimientos entre actores comunitarios y sanitarios, y la organización familiar pueden configurar condiciones favorables para una mejor calidad de vida en el tránsito hacia la muerte que considere la agencia y autonomía de la persona mayor. Esta investigación aporta una aproximación empírica situada a los cuidados de fin de vida en territorios rurales, un ámbito escasamente abordado en la evidencia científica latinoamericana, y contribuye a complejizar los debates en curso sobre la muerte asistida desde una perspectiva de geografía gerontológica^22^ y de la autonomía y agencia de las personas mayores^27^.

La transición demográfica acelerada que atraviesa América Latina y el Caribe^4^, junto al despoblamiento y envejecimiento de los territorios rurales, plantea un desafío urgente: asegurar que las personas mayores que permanecen en estas zonas tengan acceso a cuidados oportunos, integrales y culturalmente situados, incluidos los cuidados de fin de vida. Las revisiones disponibles consideran a la ruralidad como un factor de exclusión en el acceso a cuidados paliativos^57^^,^^58^, al concentrarse estos servicios en centros urbanos. Frente a esta limitación estructural, resulta crucial fortalecer la continuidad y cobertura de los programas públicos rurales, además de potenciar el papel de las comunidades rurales en la provisión y acompañamiento de cuidados.

El empoderamiento de capacidades locales, la transferencia de conocimientos entre equipos de salud y comunidades, y la valoración de los lazos familiaristas y comunitaristas de la ruralidad latinoamericana pueden constituir una base sólida para avanzar hacia modelos de cuidado que respeten la autonomía y agencia de las personas mayores. Ello implica comprender el fin de la vida no solo como un momento clínico, sino como un proceso vital atravesado por relaciones de cuidado, derechos humanos y dignidad.

En Chile, en el marco de procesos sociopolíticos en los que la alta demanda de cuidados es evidente^23^, recoger e integrar las necesidades y realidades de las comunidades rurales envejecidas es urgente. La evidencia relevada aporta información sobre los procesos de envejecimiento de territorios cuya ruralidad es diversa y adversa por sus características geográficas. En estos territorios, donde es palpable el cruce entre envejecimiento y ruralidad, se hace relevante fortalecer tanto la investigación como las políticas públicas, dadas las cargas de dependencia y necesidades de cuidados especializados que ya están experimentando estas comunidades rurales.

Finalmente, la reflexión metodológica expone algunos sesgos. Para abordar los procesos de fin de vida de personas mayores desde la investigación cualitativa, se requiere, sin duda, preparación y competencias tanto técnicas como éticas, lo que también debiese incluir a las y los profesionales de la salud. Las preguntas en torno a la muerte son complejas, no solo para las personas que están atravesando procesos de fin de vida y quienes las cuidan y acompañan, sino para quienes la investigan. En contextos epidemiológicos y patrones de mortalidad en Chile y América Latina que requieren evidencia sociosanitaria empírica y situada para dar cuenta de la vivencia de la muerte en la vejez, este estudio puede ser un aporte para el abordaje del fin de vida en la población mayor.
